# Investigations on the Effect of Layers’ Thickness and Orientations in the Machining of Additively Manufactured Stainless Steel 316L

**DOI:** 10.3390/ma14071797

**Published:** 2021-04-05

**Authors:** Abdulmajeed Dabwan, Saqib Anwar, Ali M. Al-Samhan, Abdullah AlFaify, Mustafa M. Nasr

**Affiliations:** Industrial Engineering Department, College of Engineering, King Saud University, PO Box 800, Riyadh 11421, Saudi Arabia; sanwar@ksu.edu.sa (S.A.); asamhan@ksu.edu.sa (A.M.A.-S.); aalfaify@ksu.edu.sa (A.A.); mhayal@ksu.edu.sa (M.M.N.)

**Keywords:** additive manufacturing, laser-powder bed fusion, SS 316L, part orientations, layer thickness, surface roughness, machining

## Abstract

Laser-powder bed fusion (L-PBF) process is a family of modern technologies, in which functional, complex (3D) parts are formed by selectively melting the metallic powders layer-by-layer based on fusion. The machining of L-PBF parts for improving their quality is a difficult task. This is because different component orientations (L-PBF-layer orientations) produce different quality of machined surface even though the same cutting parameters are applied. In this paper, stainless steel grade SS 316L parts from L-PBF were subjected to the finishing (milling) process to study the effect of part orientations. Furthermore, an attempt is made to suppress the part orientation effect by changing the layer thickness (LT) of the parts during the L-PBF process. L-PBF parts were fabricated with four different layer thicknesses of 30, 60, 80 and 100 μm to see the effect of the LT on the finish milling process. The results showed that the layer thickness of 60 μm has significantly suppressed the part orientation effect as compared to the other three-layer thicknesses of 30, 80 and 100 μm. The milling results showed that the three-layer thickness including 30, 80 and 100 μm presented up to a 34% difference in surface roughness among different part orientations while using the same milling parameters. In contrast, the layer thickness of 60 μm showed uniform surface roughness for the three-part orientations having a variation of 5–17%. Similarly, the three-layer thicknesses 30, 80 and 100 μm showed up to a 25%, 34% and 56% difference of axial force (Fa), feed force (Ff) and radial force (Fr), respectively. On the other hand, the part produced with layer thickness 60 μm showed up to 11%, 25% and 28% difference in cutting force components F_a_, F_f_ and F_r_, respectively. The three-layer thicknesses 30, 80 and 100 μm in micro-hardness were found to vary by up to 14.7% for the three-part orientation. Negligible micro-hardness differences of 1.7% were revealed by the parts with LT 60 μm across different part orientations as compared to 6.5–14% variations for the parts with layer thickness of 30, 80 and 100 μm. Moreover, the parts with LT 60 μm showed uniform and superior surface morphology and reduced edge chipping across all the part orientations. This study revealed that the effect of part orientation during milling becomes minimum and improved machined surface integrity is achieved if the L-PBF parts are fabricated with a layer thickness of 60 μm.

## 1. Introduction

Stainless steel (SS) 316L is one of the most common stainless steels in the engineering and medical field due to its excellent corrosion and oxidation resistance characteristics [1]. It is also used because of its combination of strong mechanical characteristics [2]. In particular, SS 316L is used as an implant material in the medical field as a result of its unique property of high corrosion resistance and biocompatibility [3]. The required corrosion resistance and ductility of the SS 316L fit the most difficult industrial applications. SS 316L in combination with Laser-powder bed fusion (L-PBF) can support individualized implants or prostheses at low cost [4]. Since these implants communicate with the human body, particular attention should be paid to their surface characteristics. However, L-PBF parts usually suffer with poor surface quality [5]. Improvement of surface traits of the L-PBF produced SS 316L parts by machining can significantly enhance their quality. Therefore, a comprehensive work is required on the machinability of SS 316L L-PBF parts to achieve the desired surface quality.

As the demand for fast and cheaper products grows, easy manufacture of components from metal powders without tools and dies is becoming increasingly desirable [6]. L-PBF can meet these demands. L-PBF is a powder bed-based AM technology, allowing the processing of various types of materials starting from a 3D CAD model to the production of metallic and non-metallic materials. The method of fusing a sequence of successive layers of metallic or composite powder materials upside down creates complex three-dimensional components [7]. In this process, a high-energy laser is used to selectively fuse thin powder layers. L-PBF technology, therefore, possesses a great potential to manufacture high-quality engineering metal components that are not easily produced using other traditional processing techniques [8]. L-PBF process is used to support a large range of applications, including aerospace turbine blades [9], bone implants [10] and automotive engine-pistons [11]. However, L-PBF fabricated parts appear to be substantially different in mechanical behavior compare to conventionally produced parts [12]. For example, some major defects remain such as poor surface finish [13]. Various techniques for reducing the surface roughness of as-built parts have been used in L-PBF. Surface re-melting and laser parameters’ adjustment, are among the most commonly used methods for reducing the roughness of as-fabricated L-PBF parts [5,14,15,16]. All of these techniques improve surface finish efficiently but still could not achieve surface roughness less than 0.8 μm to meet the demand of aerospace components and medical implants [17].

Some studies that aim to improve the surface roughness of SS 316L components by adjusting the L-PBF process parameters are reviewed in the following. Yasa and Kruth [18] reported the Ra value of the SS 316L L-PBF part decreased from 12 μm to 1.5 μm with laser re-melting. Delgado et al. [19] presented the effect of layer thickness, scan speed and building direction on the surface roughness for SS 316L L-PBF parts. Their findings showed that the direction of the building greatly affects the quality of the part. Ali et al. [20] performed a comparative study on the spatter and virgin powder particles for SS 316L L-PBF parts. The parts printed from the spatter powder particles show a 28% and 15% increase in surface roughness for cylindrical and tensile samples, respectively. Gupta et al. [21] studied the effect of layer rotation (0°, 45°and 90°) on microstructure, grain size, surface topography, relative density and mechanical behavior of Al-Si-10Mg L-PBF parts. Their findings showed that the grain size, residual stresses and surface roughness (77.02%) values reduced with increase in layer rotation angle. Alrbaey et al. [13] focused on enhancing the final surface texture of SS 316L parts during the L-PBF process. The components were developed with different slope angles to achieve a range of surface roughness between 8 and 20 μm. A custom-made hybrid laser re-cladder was used for the re-melting process. The findings showed that surface roughness was best achieved at about 1.4 μm. Aqilah et al. [22] investigated the effect of laser strength, scan speed and hatch distance on the surface roughness for SS 316L L-PBF parts. The results showed that laser power affects the surface roughness most in relation to the speed of scanning and the hatching distance. They also employed shot peening as a post-finishing process to investigate the roughness of the developed components. Their results showed that the shot peening decreases the surface roughness of the generated parts by 33.28%.

In the previous studies, the effect of the L-PBF layer thickness has been studied on the microstructures, metallurgical behavior, ultimate tensile strength, yield strength, hardness, density and surface roughness, as discussed in the following. Dadbakhsh and Hao [23] studied the microstructure of Al/5wt% Fe2O3 L-PBF parts in different laser powers and scanning speeds under the influence of various layer thicknesses (LTs). They found that the layer thickness has a strong influence on the microstructure. They recommended that uniformly distributed very fine particles could be consolidated in net-shape Al composite parts by using lower scanning speed, higher laser power and lower layer thickness. Savalani and Pizarro [24] studied the effects of preheating and layer thickness on dimensional and mechanical properties of magnesium L-PBF parts. They found that the preheated paths increase the consistency of the surface (flatter and smoother surface) in case of lower thicknesses. The L-PBF parts showed surface warped and high surface roughness as the layer thickness increased. Ma et al. [25] provided a comprehensive analysis on the effects of high LTs (>200 μm) on the microstructure and performance of the 1Cr18Ni9Ti stainless steel parts through high power laser-power bed fusion (HPL-PBF) technology. They found, despite different layer-specific energies, all manufactured samples at high LTs are very close to full-density. They showed that layer thickness influences the microstructures, the metallurgical behavior and the mechanical properties (tensile strength, fracture surface analysis and hardness. of the L-PBF parts.

Shi et al. [26] investigated the effect of high layer thickness 200 μm for Ti6Al4V L-PBF parts to ensure that high density is obtained in order to generate high mechanical properties. They found that the high LT played a major role in surface roughness instead of tensile properties. They also found that the mean elongation, ultimate tensile strength and yield strength values were similar to those studies that used thin LTs; this is due to the similar microstructure and metallurgical bonding. Sufiiarov et al. [27] presented the relation between the layer thickness and mechanical properties, microstructure and relative density of the nickel-based L-PBF parts. Their findings showed that the bulk material is made up of dendritic columnar cells. The cell size depends on the LT used during the L-PBF process. Elongation at break and tensile strength also depends on the LT. The samples with a LT-30 μm show lower elongation in breakage and higher tensile strength than the samples with a LT of 50 μm. They also found, in contrast to vertical samples, that the horizontally built samples have greater strength and less plasticity. Shi et al. [28] studied the effect of a LT of 250 μm on the tensile properties and quality effect of the SS 316L L-PBF parts. They found that microstructure, mechanical properties and relative density effects are affected by the processing parameters. By remelting, they improved surface roughness and examined the effect of changing the positioning angle to enhance the parts’ quality. Nguyen et al. [29] presented Inconel 718 L-PBF parts with different LTs (20, 30, 40 and 50 μm). Their results showed higher dimensional accuracy could be achieved at the lower LTs. The lower LTs also achieved higher mechanical properties.

In comparison to alternative metal processing, including machining [30,31,32], L-PBF produced parts still face clear surface quality challenges [33]. As-fabricated L-PBF parts need to be machined to achieve better surface quality [34]. However, the literature on the machining of the L-PBF parts is limited. Kaynak and Kitay [35] presented a comprehensive work on the surface characteristics of SS 316L L-PBF parts. They achieved the improvement of the surface by post-processing operations including finish machining (turning process), vibratory surface finishing and drag finishing. The lowest surface roughness (~Ra = 1.5 μm) was achieved by the turning operation; however, some surface integrity issues were also reported. Surface integrity issues could be because of fact that they used dry machining. Singla et al. [36] considered various post processing techniques (shot peening, heat treatments, hot isostatic pressing and sand blasting) by different researchers on Ti6Al4V L-PBF part in order to enhance the mechanical and metallurgical properties. They found that combination of post processing methods is worth exploring for reducing the defects and improving the performance of Ti6Al4V L-PBF part in different engineering applications. Struzikiewicz et al. [37] studied the effect of feed rate, cutting speed and depth of cut on surface roughness of SS 316L L-PBF parts through turning process parameters. The results showed that the feed rate has the most impact on surface roughness. Matras [38] improved L-PBF produced AlSi10Mg parts’ surface roughness by subjecting them to the milling process. The obtained surface roughness was Ra = 0.14 μm and Rz = 1.1 μm for face milling operation. Marimuthu et al. [39] reduced the surface roughness of Ti6Al4V L-PBF part from 10.2 μm to 2.4 μm by utilizing a laser polishing process. Balan et al. [40] reduced the surface roughness of maraging steel direct metal laser sintering (DMLS) parts by about 87% in grinding operation by using cryogenic condition over dry grinding. Dabwan et al. [41] investigated the effect of the finishing process on the surface roughness, surface morphology and cutting force for Ti6Al4V EBM parts. The obtained surface roughness was Sa = 0.11 μm. Kaynak and Tascioglu [42] presented the effect of the finish machining process on surface roughness, microhardness and XRD analysis of Inconel 718 L-PBF parts. It is reported that surface finish (Ra) was reduced by 92%, while the hardness increased about 16%. However, no previous work has focused on achieving the L-PBF 316L SS parts surface quality by using a machining process with an emphasis on the L-PBF parts orientations with regard to the tool feed direction (TFD). Other studies have been reported on the milling of electron beam melted (EBM) γ-TiAl and Ti6Al4V parts by [43,44]. They reported that the different EBM part orientations during machining can generate various surface roughness even with the same machining parameters. In this study, it is proposed to study the effect of the L-PBF layers/part orientations with respect to the TFD during milling. Furthermore, an attempt is made to reduce the effect of the part orientations during machining by tuning the layer thickness (LT) during the L-PBF process.

From the previous studies, it can be noted that the machining of L-PBF components is important in order to obtain the required surface quality in several applications. Although several researchers have optimized L-PBF parameters to manufacture SS 316L parts, still the poor surface roughness of Sa = 6–23 μm has been reported. At the same time, it is difficult to machine the L-PBF components because of the directional properties resulting from the orientations of the melted layers. This research aims to improve the surface quality of the SS 316L L-PBF parts by the milling process. For this purpose, the SS 316L L-PBF parts are produced with different LTs 30, 60, 80, 100 μm to study the effect of LT on machining behavior. In addition, the SS 316L L-PBF parts’ orientations are taken into account during machining. The effect of process parameters of milling (feed rate and cutting speed), L-PBF layer thicknesses and part orientations are studied on the cutting forces, surface morphology, edge chipping, surface roughness and micro-hardness.

## 2. Experimental Work

**Figure 1:**
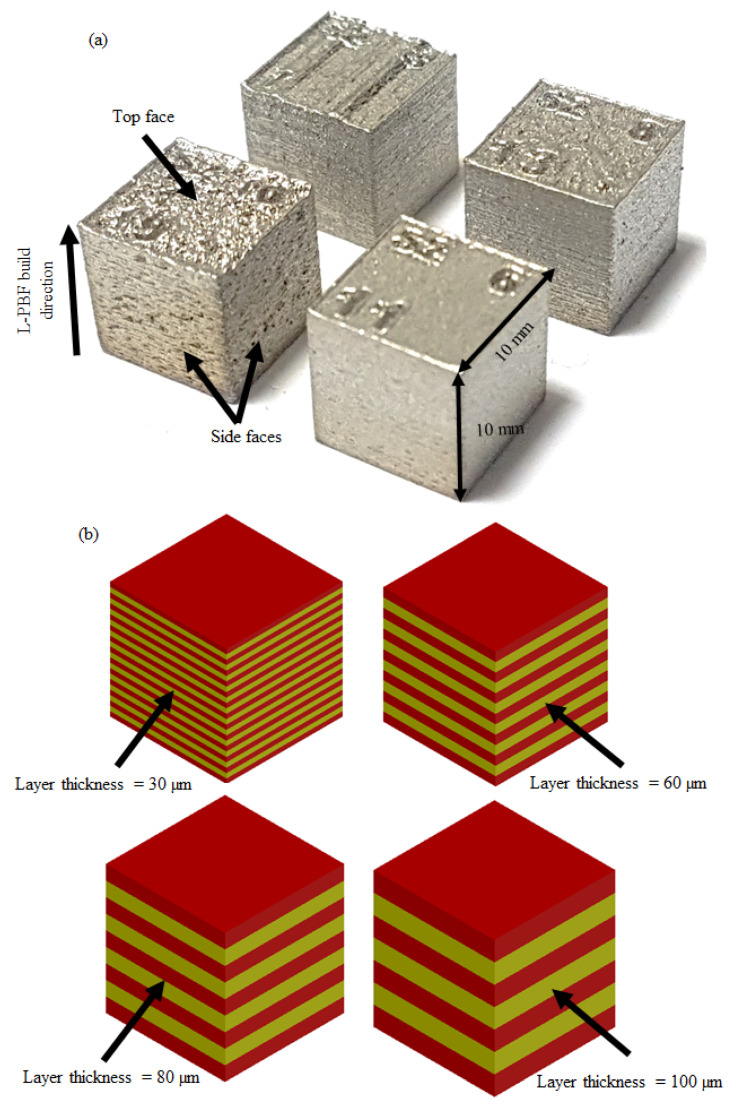
SS 316L block samples built at different layer thicknesses by L-PBF: (**a**) actual parts; (**b**) schematics of parts produced with different layer thicknesses.

SS 316L parts of 10 × 10 × 10 mm^3^ were fabricated by the L-PBF process. The powder grains of SS 316L were spherical shape with sizes in the range of 15–45 μm. The chemical composition of SS 316L powder is shown in Table 1. The L-PBF machine was a Renishaw UK AM250 model (Gloucester, UK), fitted with a 200 W laser pulsed beam. The diameter of the laser beam was 70 ± 5 μm and the build volume capacity of the unit was 250 × 250 × 300 mm^3^. The scan strategy used in this study was Meander where the scan direction of a layer rotates 67 degrees from the previous layer [45,46]. Four samples were fabricated with four different layer thicknesses (LT), as shown in Figure 1a for the actual parts, and the schematics parts, as shown in Figure 1b. The L-PBF process parameters setting to manufacture these samples is given in Table 2. To study the exclusive effect of the layer thickness on machining, the energy density was kept constant by changing the exposure time according to the relation shown in Equation (1). The L-PBF parameters listed in Table 2 were determined using previous research [45].

Energy density = (laser power)/(hatching distance × scan speed × layer thickness),(1)

The scan speed can be calculated based on the value of point distance (PD), exposure time (ET) and jump speed (JS) using Equation (2).

(2) $$\mathrm{Scan}\;\mathrm{Speed}\;\left(\mathrm{SS}\right)=\frac{\mathrm{PD}}{\mathrm{ET}+\frac{\mathrm{PD}}{\mathrm{JS}}}$$

where JS is defined by the speed of the galvanometer mirror when moving from point to point, which was kept at 5000 mm/s. Therefore, the scan speed will change as the ET changes for each layer thickness. The value of the scan speed was 1093, 564, 402 and 327 mm/s for the layer thickness of 30, 60, 80 and 100 μm, respectively. In addition, the exposure time was 50, 110, 160, 200 μs for the layer thickness of 30, 60, 80 and 100 μm, respectively.

The surface roughness of the side and top faces of the as-fabricated parts for various LTs are different, as illustrated in Table 3. The surface roughness values are still too high for many applications. For this purpose, a secondary operation is required to obtain a good surface finishing of L-PBF components. In the current study, the conventional vertical milling process is selected as a secondary operation for surface finish enhancement. The L-PBF components can be machined for the four LTs, namely LT-30, LT-60, LT-80 and LT-100, as shown in Figure 1.

**Figure 2:**
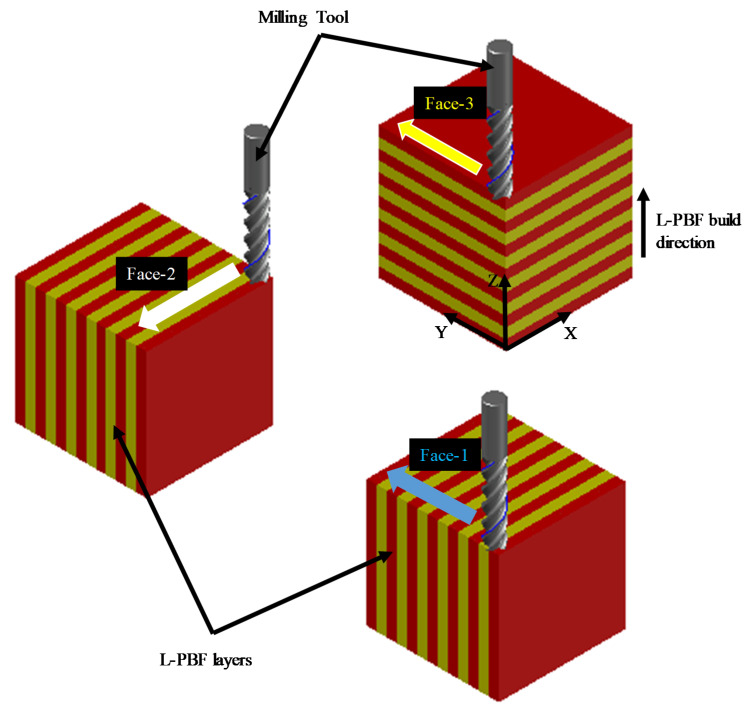
Possible L-PBF part/layers’ orientation for milling.

**Figure 3:**
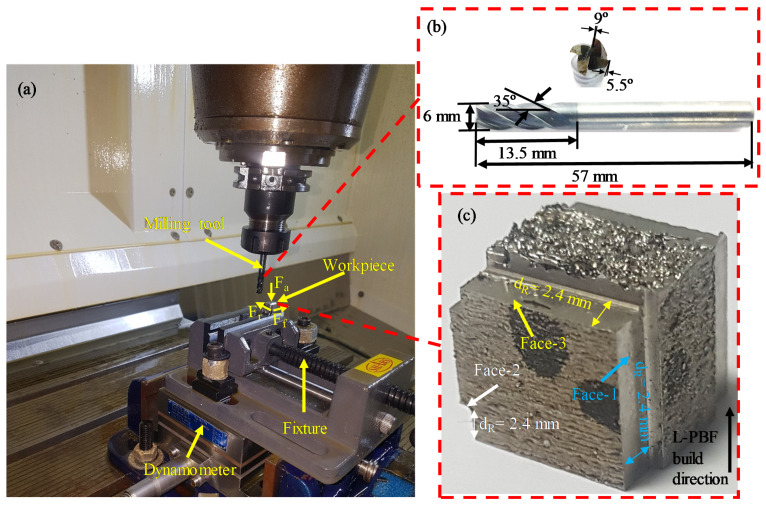
(**a**) Experimental setup; (**b**) View zoom-in of cutting tool; (**c**) View zoom-in of the machined sample.

The best orientation of the L-PBF part in terms of the TFD to achieve minimum Sa is a remarkable factor in the finishing of the additively manufactured component. The L-PBF component can be machined in three possible orientations: (1) tool feed across the layer (Face-1), (2) tool feed parallel to layers’ planes (Face-2) and (3) tool feed in a layer plane (Face-3). Figure 2 illustrates schematics of the three different orientations regard the TFD. The experimental setup is shown in Figure 3a. Figure 3b shows the geometric dimensions and parameters of the cutting tool. Figure 3c shows the three listed tool feed directions (Face-1, Face-2 and Face-3) on the L-PBF part. During milling, the samples were first subjected to a pre-machining process at a feed rate of 50 mm/min, cutting speed of 80 m/min, 6 mm tool diameter and a cutting depth of 0.4 mm. This was done to remove the irregular and rough surfaces from L-PBF parts to create smooth and uniform surfaces for subsequent finishing. Table 3 illustrates the milling process parameters to investigate the effects of L-PBF part orientations and LTs on the surface quality. This milling parameter mentioned in Table 3 falls into the range of previous SS 316L milling studies [47,48,49].

The milling experiments were performed using three-axis CNC (DMC 635 V Ecoline, DMG Mori, Oelde, Germany), which offers 8000 rpm of maximum spindle speed, 24 m/min of maximum feed speed and 1 μm of a positioning resolution. A solid carbide end mill tool with a diameter of 6 mm was used. Five responses, such as cutting force, surface roughness (Sa), micro-hardness, edge chipping and surface morphology, were measured. The Sa of the machined parts was measured using a 3D optical (Contour GT-K, Bruker Berlin, Germany) profilometer. For each of the three-part orientations (Face-1, Face-2 and Face-3) after milling, five regions of 2.2 mm × 1.7 mm with an interval of 2 mm were scanned in the middle of the machined region along the feed direction. The five readings for every orientation were averaged for the surface roughness measurements. The optical profilometer measures the 3D surfaces parameter (Sa) by means of white light interferometry. The Vision-64 software transforms the high-resolution scanned data into accurate 3D images. Later the software used ISO 25178-2 standard to calculate the 3D roughness parameter [50]. The Sa parameter is the arithmetic mean height within a sampling area as calculated by Equation (3) [51]:

(3) $$\mathrm{Sa}=\frac{1}{A}\int \underset{A}{\int }A\left|z\left(x,y\right)\right|dxdy$$

where *A* is the sampling area, *z*(*x*, *y*) number of measurement points.

The sample was mounted on a fixture located on a piezoelectric Kistler 5697A (Kistler Instruments, Kistler Corp, Winterthur, Switzerland) dynamometer during the milling process. This was done mainly to measure the axial force (F_a_), feed force (F_f_) and radial force (F_r_), see Figure 3a. The sampling frequency of the cutting force was chosen to be 1000 Hz. Durascan 10 Vickers hardness (HV) equipment (Struers A/S, Ballerup, Austria) from Austria was used with a load of 500 g and 15 s to determine the micro-hardness of the milled surfaces. For each orientation, five micro-hardness readings were taken and an average of these readings was used later. A Jeol, Tokyo, Japan (Model JCM 6000Plus) scanning electron microscope (SEM) was used to analyze the surface integrity of the samples.

## 3. Results and Discussions

### 3.1. Laser-Powder Bed Fusion of SS 316L

**Figure 4:**
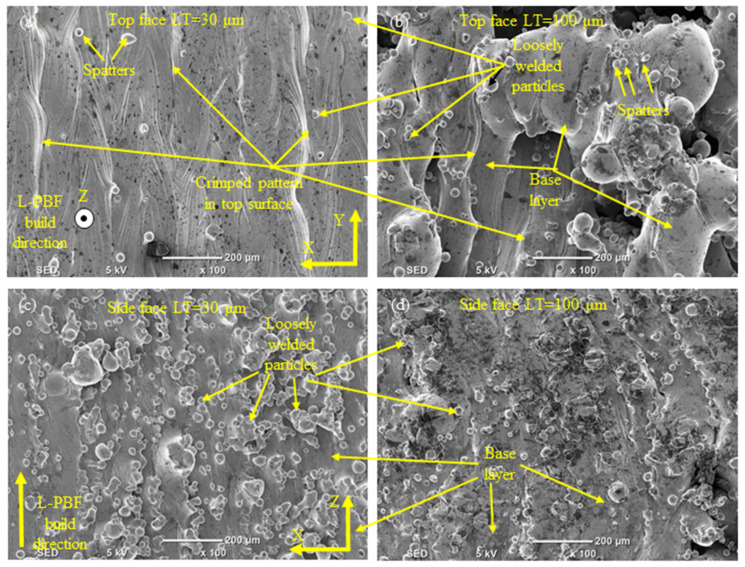
SEM of as fabricated L-PBF parts: (**a**) Top face for LT-30; (**b**) Top face for LT-100; (**c**) Side face for LT-30; (**d**) Side face for LT-100.

**Figure 5:**
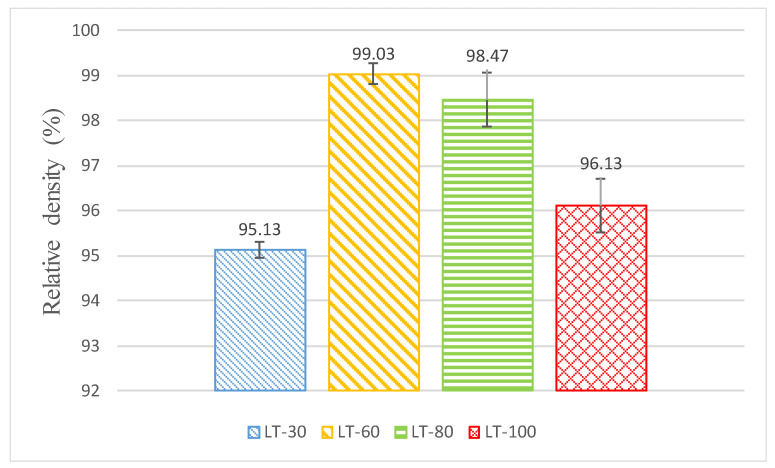
Average relative density for the bulk samples. The error bars represent ±1σ.

The SEM micrographs of the side and top of the SS 316L L-PBF parts are shown in Figure 4. The surface roughness on both sides is extremely poor, varying from 6 to 23 microns and 7 to 11 microns on the top and side face, respectively, as illustrated in Table 3. The laser beam scan marks and the distance between adjacent scanned paths have created the irregular distribution on the top face. Moreover, spatters, crimping patterns on the top and loosely welded particles further deteriorate the surface roughness on the top face. Although the same energy density was used for all the samples, still the morphology of the top face differs significantly due to the different LTs used during L-PBF. At the same time, the poor surface on the side face is mainly attributed to the loose welding of the SS 316 L powder at high temperatures at the planar edge of each deposited layer. The morphological differences in the side and top faces show that the L-PBF produced parts have directional properties. Therefore, the appropriate orientation of the L-PBF part with respect to the finishing tool in the secondary process (e.g., milling) should be carefully considered. The relative density of the bulk samples varies between 95 to 99% for different LTs, as shown in Figure 5. A similar level of densities was reported by previous authors working on L-PBF of SS 316L [52,53].

### 3.2. Surface Roughness

The surface roughness of the side and top faces of the as-fabricated parts for various LTs are different, as illustrated in Table 4.

**Figure 6:**
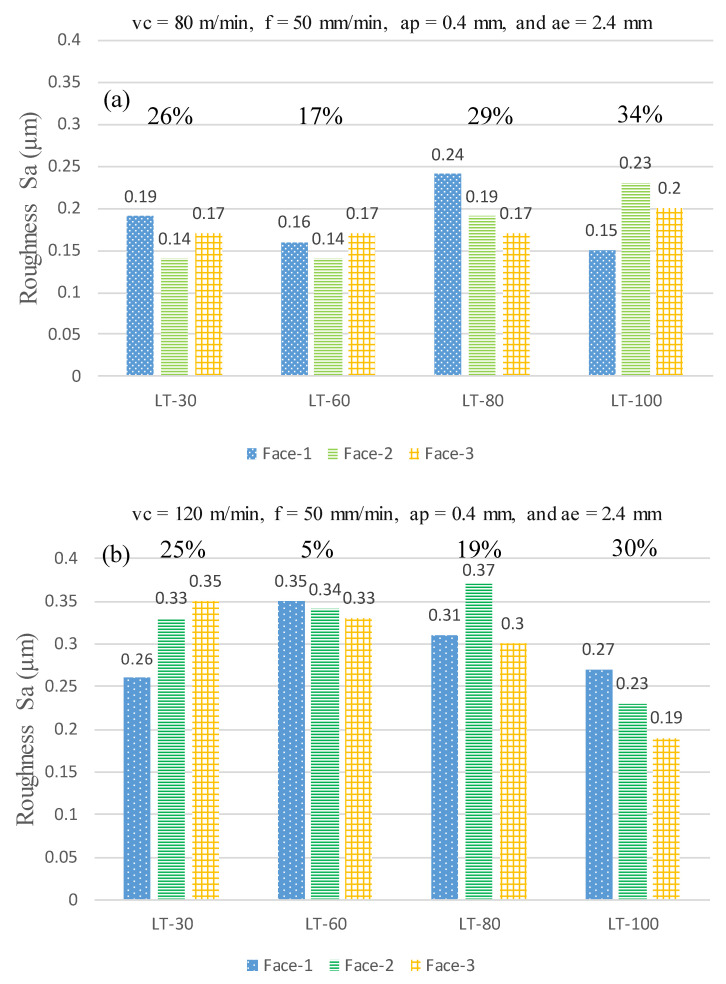
Surface roughness for three-part orientations with different LTs at machining parameters of: (**a**) vc = 80 m/min, f = 50 mm/min, ap = 0.4 mm and ae = 2.4 mm; (**b**) vc = 120 m/min, f = 50 mm/min, ap = 0.4 mm and ae = 2.4 mm. The percentages above the bars show the maximum variation among the three orientations for each LT.

**Figure 7:**
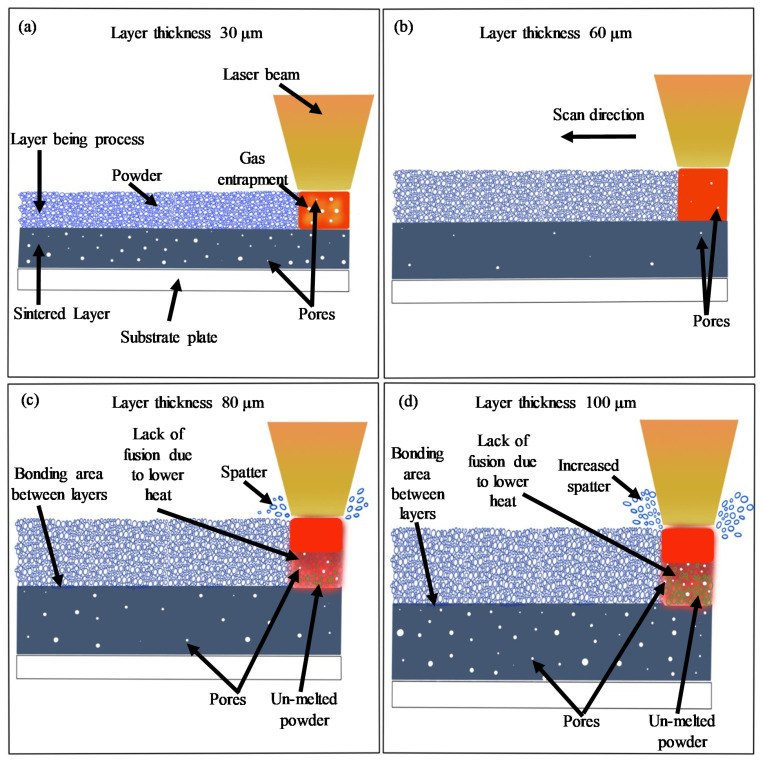
Schematic view of different LT during L-PBF process: (**a**) LT-30 μm; (**b**) LT-60 μm; (**c**) LT-80 μm; (**d**) LT-100 μm.

**Figure 8:**
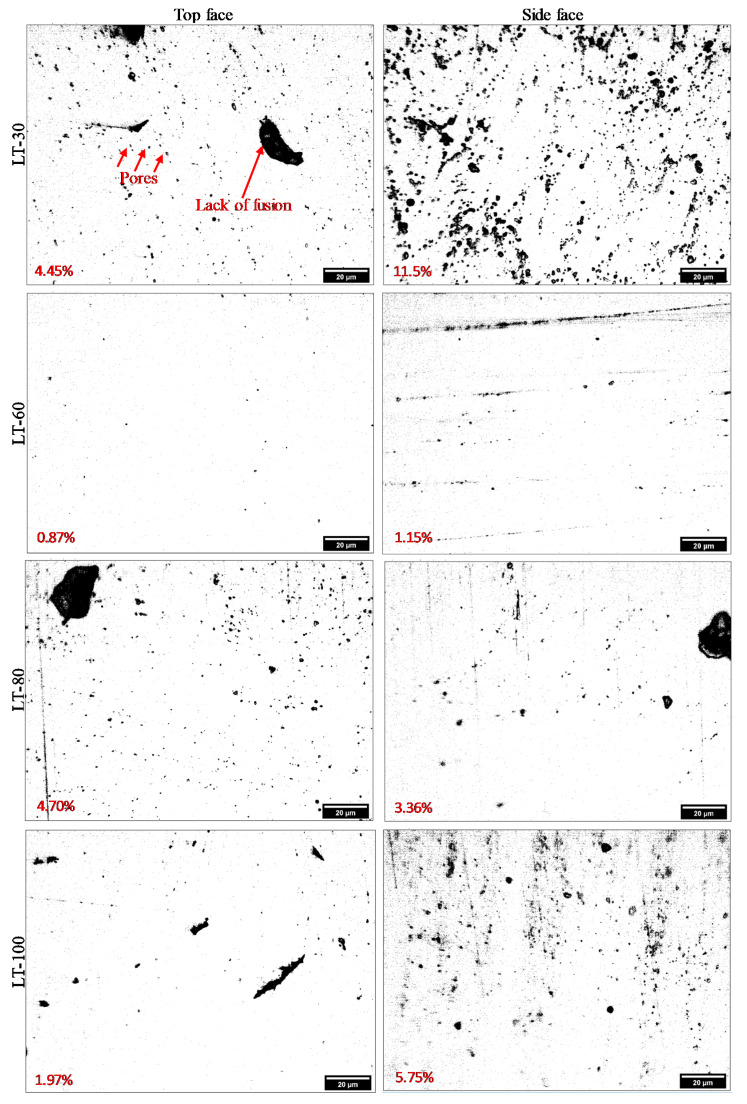
Optical image of porosity for the four LTs. The percentage in the images show the porosity.

Figure 6 shows the surface roughness (Sa) comparison of each of the three-part orientations for the L-PBF parts produced with four different LTs 30, 60, 80 and 100 μm at cutting speeds of 80 m/min and 120 m/min. There is a difference in the surface roughness among the Face-1, Face-2 and Face-3 for the three LTs 30, 80 and 100 μm with the maximum variations ranging from 19% to 34%, as shown in Figure 6. In contrast, the maximum variation in roughness among different part orientations ranges from 5% to 17% for LT-60, as shown in Figure 6. The reasons for this behavior are discussed as follows. A schematic is presented in Figure 7 to show different phenomena occurring during the L-PBF process due to the variation of the LT while keeping the same energy density. The denudation [54], recoil pressure [55] and degree of spatter [56] lead to changes in the porosity and consequently vary the strength of L-PBF parts with different LTs [27]. The applied energy density is very high for LT-30 leading to high porosity due to gas entrapment and denudation, as explained by [57,58] and shown in Figure 7a. At the same time, the same energy density is appropriate for LT-60 resulting in low porosity, as shown in Figure 7b. In the LT-80, the energy density becomes insufficient to penetrate into the powder layer to the pre-solidified layers due to the thermal loss to voids, which leads to un-melted powder particles and lack of fusion/joining with the previous layer, as shown in Figure 7c. The degree of lack of laser penetration is further increased in the case of LT-100 resulting in an increased lack of fusion (welding between layers/poor connectivity) and higher porosity and voids [45,59], as shown in Figure 7d. This is because varying surface roughness and porosity is encountered for L-PBF parts produced with different LTs [60,61]. The differences in porosity and lack of fusion can be noted between the side and top faces for LTs 30, 60, 80 and 100 μm, as shown in Figure 8. It should be noted that the porosity percentages shown in Figure 8 represent the porosity level only for the cross-section under observation. Significantly lower porosity and negligible lack of fusion are observed in the case of LT-60 as compared to the other LTs, which results in improved machining. Furthermore, the difference between the side and top faces are also minimum for LT-60, which results in least variation while machining across different part orientations as compared to the other LTs (see Figure 6).

The surface roughness (Sa) is dependent on several factors, such as layer thickness, cutting speed and layer orientation (Face-1, Face-2 and Face-3). However, these factors have contradicting effect on Sa, i.e., one factor might be increasing the Sa but the other might result in lowering the Sa. Therefore, due to the complicated relation of the effect of these parameters no fixed trend is achieved for Sa in Figure 6. For example, the increase in the cutting speed promotes the temperature rise during machining which leads to the thermal softening phenomenon in the material being machined. The speed V = 80 m/min leads to moderate thermal softening of the material and makes it easier to machine, while further increase in the temperature at V = 120 m/min adversely affects the surface quality due to excessive material softening [62,63]. However, when the Face-1 in Figure 6a is compared with Face-2 in Figure 6b for the LT-80, there is a difference in the layer orientations tool is passing through which leads to difference in the cutting forces. Higher cutting forces are generated when the tool feed is along Face-1 and lower cutting forces are generated when the tool is fed across Face-2. It should be noted that cutting forces also affect the surface roughness as reported by [44,64]. Therefore, due to cutting forces and cutting speed changing simultaneously along with complicated distribution of the porosity in the L-PBF parts, no fixed trends are observed for Sa in Figure 6. Despite this, it can still be observed in Figure 6 that even after machining at different cutting speeds and part orientations, the lowest levels of variations (17% and 5%) are observed in Sa for LT-60.

### 3.3. Cutting Force

**Figure 9:**
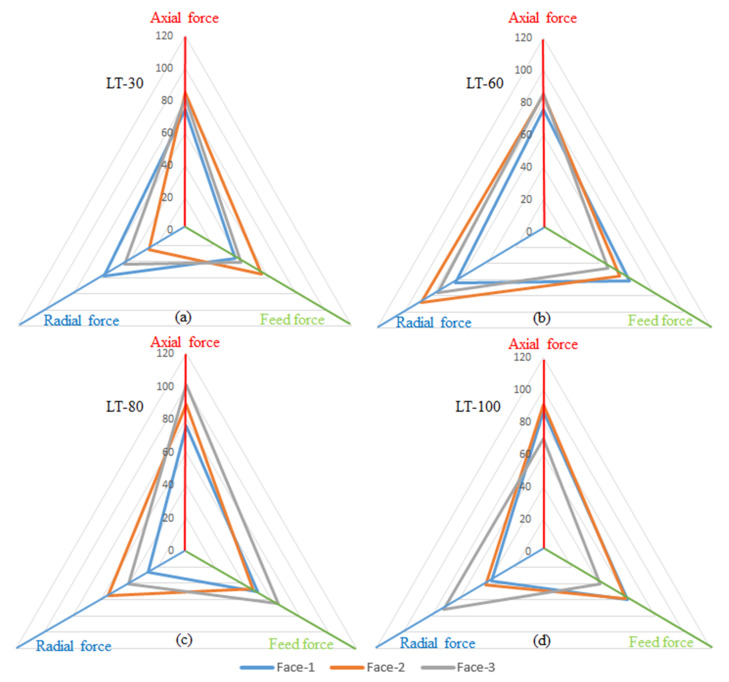
F_a_, F_f_ and F_r_ forces for Face-1, Face-2 and Face-3 measured while milling for the four LTs: (**a**) LT-30 μm; (**b**) LT-60 μm; (**c**) LT-80 μm; (**d**) LT-100 μm at vc = 80 m/min, f = 50 mm/min, ap = 0.4 mm and ae = 2.4 mm.

Figure 9 shows the maximum axial force (F_a_), feed force (F_f_) and radial force (F_r_) comparison of each Face-1, Face-2 and Face-3 of SS 316L parts with four different LTs. In Figure 9a,c,d there is a difference among the Face-1, Face-2 and Face-3 with the three-LTs 30, 80 and 100 μm. In contrast, there is a small difference in cutting forces for Face-1, Face-2 and Face-3 for the LT-60, (see Figure 9b). For LT-30, the differences of the three-part orientations are almost 12%, 34%, 56% for F_a_, F_f_ and F_r_, respectively. In the case of LT-80, the differences of the three-part orientations are almost 25%, 28%, 51% for F_a_, F_f_ and F_r_ respectively. Similarly, in the LT-100 the differences of the three-part orientations are almost 23%, 33%, 48% for F_a_, F_f_ and F_r_ respectively. Nevertheless, for the LT-60 the differences are almost 11%, 25%, 28% for F_a_, F_f_ and F_r_, respectively. The higher porosity in LT-30 leads to discontinuous machining along the tool feed direction which affects the cutting force. Moreover, the differences in porosity in the top and side faces lead to variation in the cutting forces with the part orientations. In contrast, the lower porosity in LT-60 and negligible differences in the side and top faces lead to uniform cutting force across different part orientations. The differences in cutting force for LT-80 and LT-100 also happened due to differences in porosity in top and side faces, as shown in Figure 9. Furthermore, the change in tensile strength of the L-PBF parts with a change in the LT was also reported by Sufiiarov et al. [27], which can cause difference in the machining forces for different LTs. They also established that the horizontally build samples show a change in strength properties compared to the vertically build samples [27], which advocates the variation in the cutting forces owing to part orientations.

**Figure 10:**
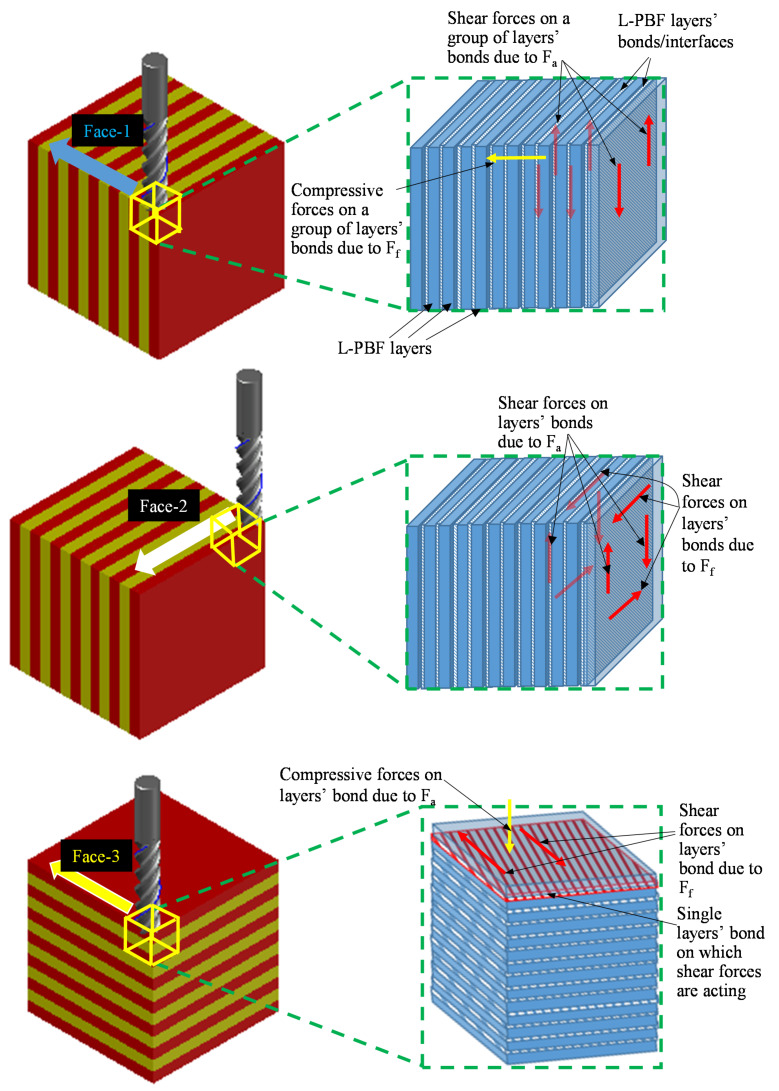
Schematic view of representation axial and feed forces as shear and compressive forces during milling process.

The differences in the machining due to the part orientations are also because of the changes in how the tool interacts with the L-PBF layers. For Face-1 orientation, the milling tool cuts a group of new bond layers under the radial depth of cut while the feed force exerts compressive forces on group of layers’ bonds/interfaces. At the same time, the axial forces exert shear forces on the plane between the adjacent layers, shown as L-PBF layer bond in Figure 10. This results in minimum tearing of the layer bonds along with the Face-1 orientation because as the tool proceeds in the feed direction, the shear forces are shifted over the new group of layers’ bonds/interfaces. However, if the tool is fed parallel to layers’ planes in Face-2, both the feed force and the axial force exert shear forces on the group of the layers’ bonds, as shown in Figure 10. However, unlike in the Face-1 case, the same group of the layers’ bonds sustain the shear/tearing forces until the tool leaves the workpiece in the case of Face-2. This results in more tearing of the layer bonds in the Face-2 orientation as compared to Face-1 orientation. In the case of Face-3 orientation, the tool-workpiece interaction occurs within a single L-PBF layer plane while the feed force act as shear force on the adjacent layers’ bond (as highlighted by red plane in Figure 10) and the axial force act as compressive forces. This scenario causes a maximum tearing effect because a single bond/interface between two layers is subjected to the shearing forces from the tool. In contrast, in the cases of Face-1 and Face-2, a group of layers’ bonds/interfaces were resisting the shear forces.

### 3.4. Micro-Hardness

**Figure 11:**
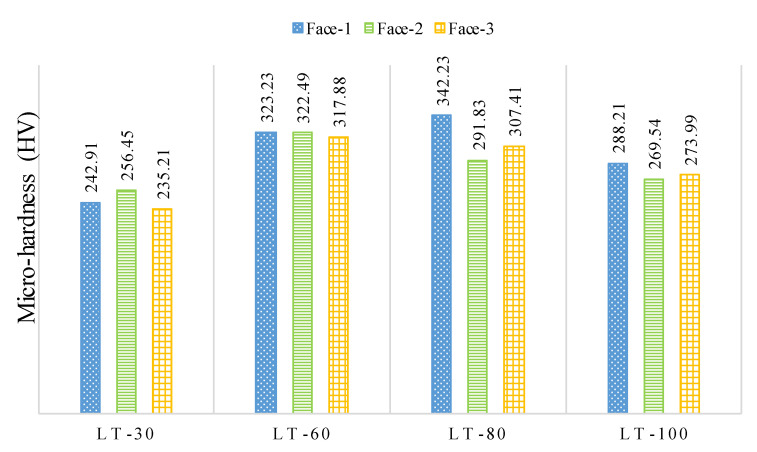
Micro-hardness measured for the three-part orientations for the four LTs on the milled surface at vc = 80 m/min, f = 50 mm/min, ap = 0.4 mm and ae = 2.4 mm.

Figure 11 displays the micro-hardness, achieved on the milled surface for the three-part orientations for the four-LTs. It can be observed in Figure 11 that there is a significant difference in the micro-hardness among the Face-1, Face-2 and Face-3 with the three-LTs 30, 80 and 100 μm. In contrast, there is a small difference among the micro-hardness for the Face-1, Face-2 and Face-3 for the LT-60, as shown in Figure 11. The significant differences of the three-part orientations are 8.3%, 14.7%, 6.5% for LTs 30, 80 and 100 μm, respectively. This indicates that the L-PBF machining against part orientations influences the imparted hardness of the machined surface. At the same time, the differences are 1.7% for LT-60. The reason of LT-60 eliminates the effect of L-PBF part orientation concerning the milling TFD due to the enhanced fusion/consolidation of the L-PBF layers, low porosity and lesser differences between the side and top faces [45,59]. Since the same energy density was used for all LTs. However, the same energy density is very high for LT-30 μm and just appropriate for LT-60 μm and becomes insufficient for 80 and 100 μm LTs.

### 3.5. Surface Morphology

**Figure 12:**
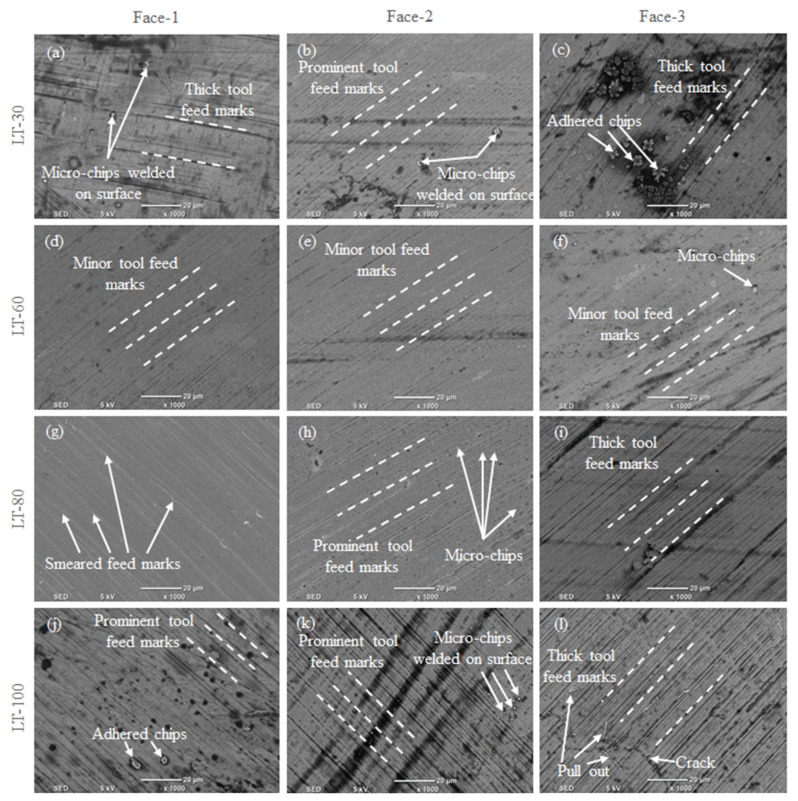
SEM image for milled surface in three orientations for the four LTs: (**a**–**c**) LT-30 μm; (**d**–**f**) LT-60 μm; (**g**–**i**) LT-80 μm; (**j**–**l**) LT-100 μm at vc = 80 m/min, f = 50 mm/min, ap = 0.4 mm and ae = 2.4 mm.

Figure 12 presents the SS 316L L-PBF surface morphology, milled under three-part orientations for the four-LTs. The micrographs of the scanning electrons show different tool feed marks, the adhesion of chips to the machined surfaces, micro-pit and smeared feed marks. For LT-30 the tool feed marks are thick and there are micro-chips welded on the milled surface for Face-1, as shown in Figure 12a. Moreover, Figure 12b shows the micro-chips welded on the milled surface and prominent tool feed marks for Face-2. For Face-3, Figure 12c shows the thick tool feed marks and adhered chips on the milled surface. In the case of LT-60, for all part orientations (Face-1, Face-2, Face-3) the tool feed marks are minor, as shown in Figure 12d–f. In the case of LT-80, the smeared feed marks are exhibited for Face-1, as shown in Figure 12g. At the same time, the prominent tool feed marks and micro-chips are shown in Figure 12h for Face-2. Furthermore, for Face-3 the thick feed marks are exhibited in Figure 12i. In the case of LT-100, there are prominent tool feed marks and adhered chips in Face-1 as shown in Figure 12j. In addition, Face-2 has micro-chips and prominent tool feed marks, as shown in Figure 12k. The cracks and pull out are exhibited in Face-3, as well as the thick feed marks as shown in Figure 12l.

Regarding Face-1, the bonds between group of layers 30, 60, 80 and 100 μm are across to the tool feed direction. The layer bonds of the different layers 30, 60, 80 and 100 μm covered under the ae of 2.4 mm (2400 μm) collectively constituted a solid base for milling resulting in different surface morphology. In Face-2, there are differences in the thickness of the layers 30, 60, 80 and 100 μm along the build direction. The layer bonds of the multiple layers are covered under the ae of 2.4 mm (2400 μm), while a tool pass removes around 80, 40, 30 and 24 layers for LTs 30, 60, 80 and 100 μm, respectively, in this way. For Face-3, the L-PBF spread layers are in-plane to the TFD. The LTs of powder melted by laser was set to different values 30, 60, 80 and 100 μm during the SS 316L L-PBF, while the cutting depth (ap) was fixed at 400 μm during the machining process. A tool cut almost 13, 6, 5, 4 layers for LTs 30, 60, 80 and 100, μm respectively. However, the layer being cut exerts shear and compressive forces on the underneath layer or layer bond, which affects the milled surface concerning layer thickness.

### 3.6. Edge Chipping

**Figure 13:**
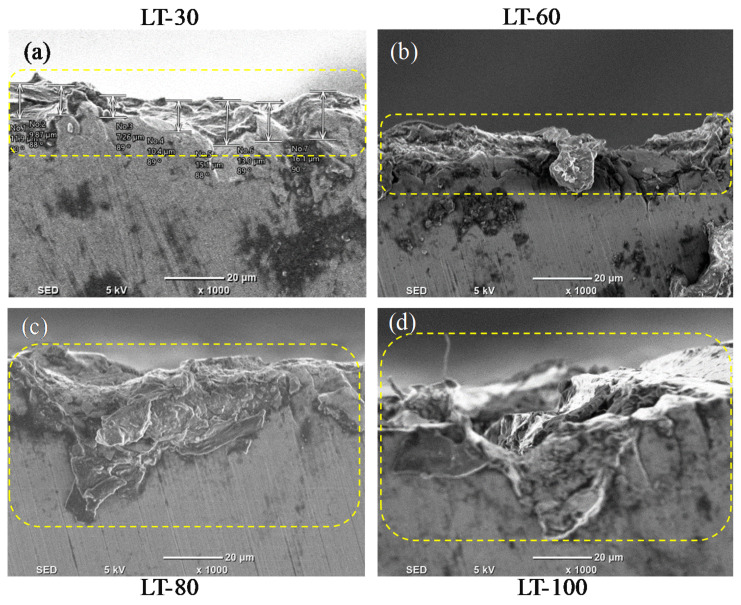
Edge chipping of milled surface in Face-1 for different LTs: (**a**) LT-30 μm; (**b**) LT-60 μm; (**c**) LT-80 μm; (**d**) LT-100 μm at vc = 80 m/min, f = 50 mm/min, ap = 0.4 mm and ae = 2.4 mm.

During the machining of L-PBF components, it was found that the milling tool causes the material to be chipped off on the edges of the part. There are differences in the edge chipping for the three orientations (Face-1, Face-2 and Face-3). Figure 13 presents the side edge chipping of the L-PBF parts milled in Face-1 for the four-LTs. The micrographs of scanning electron generated after machining for the four-LTs are different with regard to side chipping. In Figure 13a, the LT-30 presents the side chipping is found to be low. Moreover, In Figure 13b the LT-60 present that the side chipping is found to be low also. At the same time, In Figure 13c,d, the LT-80 and LT-100 present the side chipping which is found to be high.

**Figure 14:**
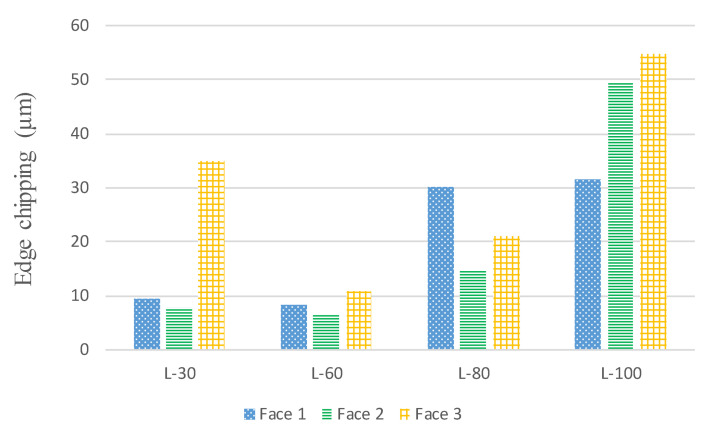
Side chipping for three-part orientations with different LTs at a. vc = 80 m/min, f = 50 mm/min, ap = 0.4 mm and ae = 2.4 mm.

Figure 14 shows the difference among the three-part orientations with the four-LTs which shows that the LT-60 has less difference between the three-part orientations in side chipping. For the results presented in Figure 14, it can be observed that the same processing parameters (feed rate, radial cutting depth, cutting speed, cutting depth) were used, still, there is high variability among the side chipping values recorded for three orientations in the four-LTs. The side chipping is low in LT-30 and LT-60 due to strengthening the bonding of the layer because of the applied heating. At the same time, the applied heating is not sufficient for bonding the layer in LT-80 and LT-100 which contributes to higher side chipping regard tool feed direction.

## 4. Conclusions

This paper presents a study on improving the surface quality of the SS 316L components produced by L-PBF with different LTs, while also taking into account the part orientations effect during machining. It is found that with regard to the tool feed direction, even under the same parameters, L-PBF parts have different machining performances for the considered component orientations (Face-1, Face-2 and Face-3) and LTs 30, 60, 80 and 100 μm. The results revealed that the LT of 60 μm is more appropriate for L-PBF parts for producing uniform surface quality after machining for all part orientations. The key findings from the current work are given below:

- SS 316L L-PBF parts revealed high surface roughness values on the top and side faces despite using the different layer thickness (LTs).
- Regarding the surface roughness after machining, in the case of the layer thicknesses 30, 80 and 100 μm, variation ranges from 19% to 34%. Meanwhile, in the case of the layer thickness 60 μm a uniform surface roughness was produced for all the Faces/part orientations with variation ranging from 5% to 17%.
- For cutting forces, in the case of the layer thickness of 30 μm, the differences are almost 12%, 34%, 56% for F_a_, F_f_ and F_r_, respectively. Meanwhile, for LT 80 μm, the variations among F_a_, F_f_ and F_r_ are almost 25%, 28% and 51%, respectively. In addition, for the layer thickness 100 μm the differences are almost 23%, 33% and 48% for F_a_, F_f_ and F_r_, respectively. Nevertheless, for the layer thickness of 60 μm, the differences are found to be the lowest at 11%, 25% and 28% for F_a_, F_f_ and F_r_, respectively.
- After machining of the L-PBF parts produced with LT 60 μm, the micro-hardness differences among all the Faces were reduced to an insignificant of 1.7%. On the contrary, the micro-hardness variations of 8.3%, 14.7%, 6.5% were observed after milling across different faces for LTs 30, 80 and 100 μm, respectively.
- For LTs 30, 80 and 100 μm, the machined L-PBF parts showed varying surface morphology for different Faces with indications of welded micro to macro-sized chips and fluctuating minor to thick tool feed marks. In contrast, only minor tool feed marks and some micro-redeposited chips were observed after milling of the LT 60 μm parts for any Faces/part orientations.
- The side edge chipping is found to be high for the two-layer thicknesses 80 and 100 μm. Meanwhile, the layer thickness of 30 and 60 μm present low side edge chipping.
- Overall, the results indicate that the L-PBF parts produced with LT 60 μm present better machinability in terms of improved surface finish and surface integrity. In addition, the Face-1 orientation must be preferred while finishing the L-PBF parts.

## Figures and Tables

**Table 1:** SS 316L powder of chemical composition.

Element	Wt. (%)
Cr	17.50–18.00
Ni	12.50–13.00
Mo	2.25–2.50
Mn	≤2.00
Si	≤0.75
Cu	≤0.50
N	≤0.10
O	≤0.10
P	≤0.025
C	≤0.030

**Table 2:** L-PBF process parameters used for manufacturing 316L SS.

L-PBF Parameters	Unit	Values
Point distance	μm	70
Laser power	W	200
Hatching distance	μm	120
Energy density	J/mm^3^	50
Layer thickness	μm	30, 60, 80, 100

**Table 3:** Process parameters of L-PBF part.

Parameters	Symbol	Value
Cutting speed	vc	80, 120 m/min
Feed rate	f	50 mm/min
Depth of cut	ap	0.4 mm
Radial depth of cut	ae	2.4 mm
Layer thickness	LT	30, 60, 80, 100 μm
Tool feed direction	TFD	Face-1, Face-2, Face-3

**Table 4:** Sa of as-fabricated parts for different layer thickness.

	LT-30	LT-60	LT-80	LT-100
Top side	12.37	6.05	9.63	23.09
Side face	11.58	7.54	8.28	9.41

## Data Availability

Not applicable.
